# Social prescribing: Exploring general practitioners' and healthcare professionals' perceptions of, and engagement with, the NHS model

**DOI:** 10.1111/hsc.13935

**Published:** 2022-07-23

**Authors:** Coco Moore, Peter Unwin, Nick Evans, Frances Howie

**Affiliations:** ^1^ School of Allied Health and Community The University of Worcester Worcester UK; ^2^ School of Science and the Environment The University of Worcester Worcester UK; ^3^ Three Counties School of Nursing and Midwifery The University of Worcester Worcester UK

**Keywords:** community prescription, link worker, primary care, public health, social prescribing

## Abstract

Social prescribing (SP) has rapidly expanded over recent years. Previously a bottom‐up, community‐led phenomenon, SP is now a formal part of structured NHS policy and practice. This study was designed to ascertain how general practitioners and other primary healthcare professionals (HCPs) within one clinical commissioning group (CCG) perceive and engage with this new NHS model. The research comprised an online survey distributed to HCPs within a predominately rural, English CCG between June and August 2021. Qualitative data were gathered and analysed using reflexive thematic analysis. Positive portrayals of SP were found, although definitions and perceptions varied greatly. Many HCPs reported high levels of engagement with SP services; yet referral rates appeared to remain significantly lower than the previously estimated 20% of primary care attendees referred for social reasons. Moreover, 96% of HCPs reported signposting patients directly to community or external services, rather than referring them to SP. This signposting, which has been positioned as a model of SP, reflects engagement with SP in practice, which is likely to have pre‐dated the introduction of the fuller NHS model. HCPs may be unaware that this could be classed as a social prescription, and this type of SP remains uncaptured within NHS statistics. These results indicate an underuse of the national system set up to deliver one particular model of SP, rather than that SP does not occur. Additionally, despite national guidance issued to accompany the NHS model, practices such as referral and feedback processes, and link worker presence within practices, were not uniform even within this single CCG. Nevertheless, understanding is increasing as SP becomes embedded within primary care. The lack of consistency in referrals between practices warrants further examination in terms of equity of service choices to patients, as does the very low self‐reported referral rate to SP.

**What is known about this topic?**

- Social prescribing aims to address non‐medical issues of patients presenting to primary care services.
- The NHS model of social prescribing was introduced in the NHS Long‐Term Plan (LTP) in 2019 and is described as a key component of Universal Personalised Care within the United Kingdom.
- Little is known about recent GP and referrer engagement in the NHS SP model.

**What this paper adds?**

- An early look at the NHS SP model, which identifies significant variation in practice between NHS staff.
- Findings that overall referral rates are low despite mainly positive staff perceptions within the CCG studied.
- Recommendations that further training for clinicians locally is required to reduce variation and ensure all patients have the same opportunities for referral to SP.

## 1 INTRODUCTION

This paper explores the practice of social prescribing (SP) within a clinical commissioning group (CCG) in England. SP is a significant public health intervention, the popularity of which has been gathering momentum in recent years. We first present the definitions of SP, introduce the NHS model and discuss the existing evidence base regarding outcomes of SP interventions. We then explore the process of SP implementation within primary care.

### 1.1 Definition and background of social prescribing

SP is generally considered to be the practice of connecting primary care patients with non‐medical community sources of support (Brown et al., 2004). This non‐medical support can encompass adult education, debt advice, healthy living programmes, gardening projects, connecting patients with social groups, addressing loneliness and isolation and exercise clubs, for example. SP schemes have traditionally developed in the United Kingdom in a ‘bottom‐up’ way, resulting in diverse definitions and practices. However, the NHS Long‐Term Plan (LTP; NHS England, 2019b) formally incorporated SP into the NHS and introduced an NHS model of SP as part of its comprehensive model of personalised care (NHS England, 2019a). This NHS model of SP should be accessed equally across the United Kingdom, representing a ‘top‐down’ approach in stark contrast to what has gone before. This research provides an early investigation of this new NHS model of SP within England through a survey of primary care healthcare professionals (HCPs) within one CCG. The aim of the research was to ascertain and compare how general practitioners (GPs) within one CCG perceive, understand and engage in SP. Although the NHS LTP remains within its first few years of implementation, it is important to understand how it is being adopted and received by those responsible for its delivery. Positive engagement and perceptions would signify that the NHS model is being positively received and actively integrated into primary care practice. Alternatively, if concerns or reluctances are identified, then questions can be raised about its likely effectiveness and evidence provided of the challenges that need to be overcome with the future development of SP in primary care services.

The NHS LTP (NHS England, 2019b) firmly established SP within the NHS remit and provided funding for SP link workers under the Additional Roles Reimbursement Scheme (NHS England and NHS Improvement, 2019), which created a standard NHS model (Jani et al., 2020). According to Public Health England (2019), the UK standard model involves referral to a link worker who works with the service user to ascertain what matters to them and identify goals through shared decision‐making. The link worker then connects the person to appropriate groups or organisations that can offer emotional or practical support. This model involves referrals being made from a variety of sources, including GPs, local authorities, allied health professionals and the fire service.

### 1.2 Evidence regarding the outcomes of social prescribing

Research is rapidly increasing into such a major, yet nascent, policy initiative. Husk's (2019) editorial in the British Journal of General Practice observes that the proliferation of schemes has so far outpaced the evidence base and that results have often been contradictory. Most existing research has to date focused upon four main outcomes (or intended outcomes) of SP, which will now be reviewed in order of the significance attached to them by the NHS: promoting well‐being; reduced loneliness; relieving pressure on GPs and financial savings.

Chatterjee et al. (2018) conducted a systematic review of primary research published between 2000 and 2015, which evaluated SP schemes. Overall, the key benefits identified were improvements in mood and in psychological well‐being. Similar benefits to participants' mental well‐being were found by Moffatt et al. (2018) who conducted semi‐structured interviews with SP service users in Newcastle‐upon‐Tyne. Further supporting evidence has been published by Woodall et al. (2018) who conducted a mixed‐methods evaluation of an SP scheme in Northern England. This comprised interviews, focus groups, and a pre‐ and post‐intervention measure using the standardised Warwick‐Edinburgh Mental Well‐being Scale (WEMWBS). They reported both positive qualitative feedback and significant well‐being improvements, especially for younger participants, and an improved well‐being score for 77.5% of participants from baseline to post‐intervention. However, other research has found no significant improvement in depression scores (Carnes et al., 2017; Grant et al., 2000; Mercer et al., 2019). Additionally, it remains difficult to measure pre‐ and post‐intervention well‐being scores, as patients are often referred to SP projects alongside standard treatment methods using medication or talking therapies. This prevents the attribution of any significant differences to a specific intervention. Despite the difficulties in establishing a cause‐and‐effect relationship of SP to well‐being, research does serve to indicate that a positive association can exist between the two.

One of the main aims of SP is to target loneliness and social isolation (Carnes et al., 2017); it is the first aim presented by The Social Prescribing Network (2018). This is supported by a multitude of studies, including the following: Dayson and Bennett (2016); Moffatt et al. (2017); Woodall et al. (2018); Lynch and Jones (2019); Kellezi et al. (2019); Dayson et al. (2020); Foster et al. (2020) and Hassan et al. (2020). Dayson and Bennett (2016) carried out a mixed‐methods evaluation of the Rotherham SP pilot, finding the practice prevented isolation and increased participants' networks. Their findings were supported by service users in Moffatt et al.'s (2017) qualitative study using semi‐structured interviews with 30 long‐term condition (LTC) patients referred to an SP programme in a deprived area of Newcastle‐Upon‐Tyne. They found a decrease in social isolation and increased resilience, which was corroborated by Lynch and Jones (2019) who reported increased social support and decreased isolation for SP service users. Positive effects have also been cited as increasing relationships and social networks (Woodall et al., 2018), improving belonging and inclusion (Hassan et al., 2020), and enhancing social well‐being (Dayson et al., 2020). However, results from the only randomised controlled trail examined in published literature showed no difference in levels of social support (Grant et al., 2000). Holding et al. (2020) acknowledge that their 12‐week study was too short term to show any significant decrease in loneliness. It is unknown what the long‐term effects of SP could be on social connections, as a longitudinal study is yet to be conducted.

In terms of the crisis of pressure facing GPs, 59% of those surveyed by the Royal College of General Practitioners (2018) believed that SP could reduce their workload. This is supported by quantitative evidence from Grayer et al. (2008) who demonstrated a decrease in primary care appointments for service users to be linked with community organisations in inner‐city London. Additionally, Dayson and Bashir (2014) found patients' use of hospital resources reduced by up to one‐fifth in the 12‐month period following their referral to SP. Kimberlee (2016) reported a drop in GP appointments for service users 12 months after participating in SP in Bristol, with Carnes et al. (2017) also finding falling rates of GP usage. Loftus et al. (2017) monitored contacts with GPs and new repeat prescriptions and found no significant difference after either 12 weeks or 12 months of engagement with SP. Additionally, 53% of participants in Woodall et al.'*s* (2018) research did not feel there was a change in their GP usage caused by SP. The authors concluded that data indicating future reduction in primary care usage were inconclusive. Similarly,  Polley et al. (2017) undertook a systematic review of seven studies examining the effect of SP on GP demand. There was contradictory evidence and no firm conclusion could be established. It is also worth noting that although SP has been promoted for decreasing the pressure on GP services, both the Royal College of Occupational Therapists and the Royal College of Psychiatrists (2021) clearly state that SP should be used as an accompaniment to medical care, not as an alternative or replacement. It is also important to recognise that reducing primary care usage is not necessarily a beneficial outcome because primary care contact is valuable for symptom identification and early intervention. If service users feel discouraged from contacting their GP, it is possible that symptoms could be missed, in turn leading to poorer health outcomes. This position is supported by Woodall et al.'s (2018) participants who increased their interaction with their GP through SP service interaction claimed that they felt more aware of their own health needs. Nevertheless, as pressure on GP services continues to increase, the more preventative nature of SP could lead to reduced demand over the longer term.

Research has considered the potential cost‐savings to the NHS and social return on investment (SROI) of SP Schemes. SP has been argued to show value for money, with an SROI of £2.90:£1 (Kimberlee, 2016). That said, other evidence has claimed SP results in higher costs (Grant et al., 2000) and no significant reduction in GP usage (Loftus et al., 2017; Polley et al., 2017; Woodall et al., 2018). These discrepancies and contradictory results between studies may, in part, be due to the highly complex nature of this health intervention. The innumerable variables involved in a person's contact with their GP create a very challenging area to measure and produce reliable quantitative data. It should be cautioned that the studies scrutinised above, and indeed throughout this review, not only use different measures, but they have different patients with different backgrounds and presentations. Some are even based within different interventions because what constitutes SP has been so highly diverse prior to the introduction of the NHS model, with Kimberlee (2015) illuminating the varying and contested definitions of the practice. He organised SP models into four categories to differentiate between the diverse practices, which are signposting, SP light, SP medium and SP holistic. The activities patients are referred to by SP also differ greatly; Thomson et al. (2015) categorised these into eight forms: arts on prescription, books on prescription/bibliotherapy, education on prescription, exercise referral, green gyms/ecotherapy, healthy living initiatives, signposting/information referral and time banks. This diversity demonstrates the difficulty of comparisons. A more standard model should help reduce this problem in future research.

### 1.3 Process of implementation

This paper is particularly concerned with the relationship between GPs and SP, given the new emphasis that the NHS model now places upon it. A limited selection of previous studies have examined barriers and facilitators to GP engagement with SP (Farenden et al., 2015; Husk et al., 2020; South et al., 2008), and also the impact of the relationship between referrers and SP link workers. For example, South et al. (2008) recommended that patients be referred to a single known link worker, and Bickerdike et al. (2017) emphasised the importance of networking and communication between GPs and link workers. Maintaining a visible presence in the GP practice was argued by Fixsen et al. (2020) to be effective for promoting GP buy‐in, with referrals in their research slowing as soon as the link workers in their study were no longer regularly in the practice. However, little evidence has yet been presented to examine the NHS model and the ways in which current primary HCPs perceive, understand and engage with it. This study was carried out across primary care networks (PCNs) within one CCG based in a predominately rural area of England. It aimed to ascertain how GPs and referring HCPs view and engage with their local SP services. The findings reported here are derived from the first phase of a larger research project which also investigates the experiences of SP link workers and patients within the new NHS model.

## 2 MATERIALS AND METHODS

The principal research question was centred on how GPs and HCPs perceive, understand and engage in SP. A case study of one CCG was deemed appropriate to understand in‐depth the process, challenges and facilitators of SP in one area. Case studies are valuable for describing what is happening in a particular situation and ascertaining the complexity and situatedness of phenomena (Cohen et al., 2007). Qualitative online questionnaires were distributed to all GPs and referring HCPs within the selected CCG.

### 2.1 Survey design

The survey was designed using a combination of the factors identified through the literature search. It aimed to develop an understanding of how these, and the NHS model, were being perceived and integrated. Questions asked for demographic details, including the respondent's PCN, age, gender and how long they had been practicing in their role. They were asked to provide their definition of SP, how long they had been aware of it and ways in which their perceptions had changed in the past 2 years (since the publication of the NHS LTP). Previous literature was used to inform the investigation, such as asking if respondents were aware of their link worker, whether that link worker was based in their surgery and, if so, how often. Questions also explored referral reasons to understand if HCP referrals were congruent with the main outcome aims of SP (i.e. addressing well‐being and isolation, reducing pressure on primary care services and financial savings). Other questions explored topics of referral and feedback practices to ascertain if a standardised process exists; consideration of patients' socioeconomic status and geographical location and challenges they face with regards to SP.

### 2.2 Survey distribution

The survey was distributed via four routes designed to maximise response rates. These comprised being: featured in the SP newsletter; advertised on the local primary care intranet site; presented at the SP steering meeting; emailed to each GP surgery within the CCG, with follow‐up emails sent 2 weeks later. A small prize draw was offered to incentivise respondents. It is recognised that non‐responders may possess shared characteristics, such as dislike or apathy towards SP, or caseload pressures, which disallowed them time to participate, thereby excluding potentially valuable findings. Informed consent was gained through providing a participant information sheet and the opportunity to contact the research team with any queries. Participants were informed on how to withdraw after completing the survey, and it was made clear that identifying information would be removed to mitigate concerns that responses could reflect on participants or their surgery. Additionally, when writing results, all PCNs and surgeries were provided with pseudonyms. Full ethical approval was granted by the University of Worcester Research Ethics Board.

### 2.3 Data analysis

The method of data analysis used within this research drew upon Braun and Clarke's (2019) reflexive thematic analysis (TA). Reflexive TA emphasises the necessity of coder subjectivity rather than viewing this negatively as a form of bias. The generation of themes was a result of co‐produced knowledge between the participants, the data and the research team.

## 3 RESULTS

In total, 24 HCPs completed the survey from 11/84 GP surgeries within the CCG, across 8/15 PCNs. They comprised: 20 GPs; two Advanced Nurse Practitioners; one Advanced Clinical Practitioner; and one Practice Manager. Table 1 depicts the definitions of SP provided by respondents, displaying each respondent by number and the characteristics of SP they stated. Defining terms have not been grouped into broader categories, to avoid losing the nuances of terminology participants used or misinterpretation and the risk of assuming an unintended meaning of terminology. The table demonstrates how each respondent gave multiple definitions (or aspects) of SP, recognising the variety of interventions that SP can offer. It displays how some HCPs see SP as based in practical assistance, whereas others identified more social or emotional interventions. This contrasts with the way in which the NHS model has been theoretically conceived, whereby all stakeholders buy into one standardised and accepted definition of the practice. However, this is not a static situation in that 17 participants (70%) reported that their perception of SP had changed within the past 2 years. They stated that their knowledge of SP is increasing, coinciding with the publication of the NHS LTP. This suggests that former non‐engagers have been forced to engage or re‐evaluate their view of the contribution that SP can make to participate with this new initiative.

**TABLE 1:** Definitions of social prescribing provided by healthcare professionals

	Supporting patient	Social issues/factors	Stress	Non‐medical support/alternative	Lifestyle support/improvement	Aims to improve physical health	Plan of care	Supplements medicine	Holistic approach to health/wellbeing	Using community support/ providers	Advice or signposting	Fills a gap in services	Addresses multiple issues	Practical issues (housing, debt etc)	Addresses social isolation	Help patients manage better	New role	Mental health support	Health eating/diet/weight loss	Encourage/enable self‐management	Unsure
1	♦	♦	♦																		
2	♦			♦																	
3		♦			♦	♦															
4				♦			♦	♦	♦	♦											
5		♦			♦	♦					♦										
6						♦				♦											
7												♦	♦								
8				♦		♦					♦										
9		♦			♦	♦								♦	♦						
10																					♦
11		♦			♦	♦			♦♦		♦			♦							
12		♦				♦				♦					♦						
13	♦	♦		♦				♦													
14		♦		♦																	
15				♦												♦					
16	♦	♦												♦	♦		♦				
17		♦			♦				♦	♦			♦	♦				♦			
18					♦						♦				♦			♦	♦		
19				♦		♦								♦						♦	
20					♦				♦	♦	♦			♦				♦			
21	♦			♦						♦											
22	♦			♦	♦													♦			
23				♦					♦												
24									♦	♦											

The symbols indicate which respondent reported each definition of SP as listed in the top row.

### 3.1 Understanding and perception of social prescribing

In general, HCPs report a positive view of SP, describing it as valuable, supportive and holistic. The use of a term such as ‘holistic’ points to the adoption of the biopsychosocial model (Borrell‐Carrió et al., 2004), with HCPs addressing all aspects of health rather than purely medical concerns. Seven participants stated that their understanding of SP had increased in the past 2 years. They had gained more understanding and awareness, with two participants providing a level of explanation. For one respondent (Participant 20), case studies and examples had helped; for another (Participant 17), they replied that the SP newsletter had been useful. Despite knowledge generally increasing, as already noted, 63% still feel that they would like a deeper understanding of the practice. Responses included being unaware of some aspects of SP and a belief that greater promotion of the service was needed. There were also calls for more case studies, training and greater information about what services are available. Respondents also reported being unsure of how SP can help, who should be referred, and confusion regarding the differences between SP, lifestyle advisors and well‐being coaches. One participant believed that SP was a ‘*new service’*, and another believed SP was ‘*for older adults’*. Such findings suggest that SP has been promoted and that practitioners are aware of its existence at a general level, but that there is also a lack of awareness of detail. Further continued professional development (CPD) in this area is encouraged. Additionally, visible presence of link workers within surgery may increase HCP awareness of the service, an issue discussed in greater detail within section 3.5.

There appeared to be an inverse relationship between length of time a person had been practicing and their confidence regarding knowledge of SP. For example, Participant 10 had been practicing as a GP for 15–20 years and stated they ‘*do not know enough about the role/how they can help or who they can help’*. A shift in educational focus from biomedical to more biopsychosocial approaches is indicated. Hutchinson (2006) discussed changing medical education within UK higher education. She argued that although doctors had previously seen devolving care to other professionals as a threat, there is an increasing move in much university teaching towards instructing on multi‐professional working and understanding of alternate options for patients. This may explain why newer graduates possessed a greater understanding of the role of link workers and were more engaged with the process. As discussed above, successful SP schemes depend on suitable referrals (Holding et al., 2020) and GPs being aware of the service. Fixsen et al. (2020) found that link workers previously had continuously to remind GPs of their existence. As SP becomes more well known and embedded in practice, this may cease to be a significant issue because more primary care professionals will understand SP and what it can offer. A particular issue which appeared to inhibit referrals is a perceived lack of capacity within the SP system and delays in referred patients being seen by a link worker. An investigation of link worker capacity is indicated for future stages of the research to ascertain the accuracy of this perception.

### 3.2 Reasons for referral

A wide range of reasons is given for referral of patients to SP services. However, most HCPs focus upon one or two aspects; for example, viewing SP as primarily facilitating improvements in mental health. Surprisingly, the most common type of referral reported within the survey was referring for practical reasons, such as accessing benefits or for assistance with housing, finances and employment. Although practical support is one of the aims of SP, this is not something that has been identified as significant in previous research. There are also some answers from respondents, which demonstrate a low level of understanding of SP, such as where its purpose was expressed as for help filling in forms (Participants 5 and 21). Along with many generic answers, this domain supports the findings previously outlined that many HCPs do not yet have a comprehensive understanding of the remit and value of SP.

### 3.3 Referral process

The survey investigated referral processes because adequate and appropriate referrals are essential for the functioning of any SP scheme (Holding et al., 2020). It was previously recommended by Farenden et al. (2015) that referral processes are simple, easy to use and tailored to each GP surgery. With regards to the positive perception of referral processes, 11 participants explicitly denied any challenges in referring patients to SP. There were reports of prepopulated forms, designated proforma for referrals, ‘very quick’ forms and a central email address. Although positive, there does not appear to be one set standard form across all settings. Alternatively, some respondents felt referral practices were more challenging, such as Participant 11 who stated that ‘*the form can be a little confusing with all the boxes to tick*’, and Participant 20 who felt the *‘time taken to fill out referral form’* was a challenge regarding referring a patient to SP. Interestingly, one respondent said that patients could self‐refer, and another reported that administrators were able to refer patients directly to SP at the point of contact, skipping the step of HCP involvement. Self‐referral involves the patient directly referring themselves to the SP service, to engage with a link worker without the HCP evaluating them first within the GP surgery. This deviates from the Social Prescribing Network (2018) and Public Health England (2019) definitions of SP which include the step of a GP or frontline HCP referring the individual to the SP scheme. However, NHS England (2021) contradict this, stating ‘*When social prescribing works well, people can be easily referred to link workers from a wide range of local agencies, including general practice, pharmacies, multi‐disciplinary teams, hospital discharge teams, allied health professionals, fire service, police, job centres, social care services, housing associations and voluntary, community and social enterprise (VCSE) organisations. Self‐referral is also encouraged’*. Additionally, an internet search of ‘social prescribing self‐referral’ immediately returned many local and national options for self‐referral to schemes. Thus, this diversity is problematic to the concept of a standardised top‐down NHS model executed uniformly across the United Kingdom.

### 3.4 Professional engagement

The positive engagement of HCPs is essential for the success of SP schemes for two reasons. First, SP itself relies upon adequate and appropriate referrals into the service (Holding et al., 2020). Second, the way in which an HCP interacts with the patient when recommending a referral to SP has been claimed to impact significantly on how the referral is received and how the patient engages with it (Husk et al., 2020). The survey data show that 23 of 24 participants (95.8%) had previously referred patients into SP. Respondents were asked how often they referred to SP and responses were sorted into categories, which can be seen in Table 2. Responses ranged from referring multiple times per week to less than once a month, with the most common being around once a fortnight. This is a valuable insight because, assuming the average HCP sees 41 patients per day in the UK (Campbell, 2019; Merrifield, 2019), a five‐day working week would mean they see 205 patients a week or 820 per month. Referring two of these 820 patients per month would be a 0.24% referral rate. This is significantly less than the estimated 20% of GP appointments where the nature of the patient's consultation is rooted in social issues (Husk, 2019). It is noted that these are not verified statistics and the number of patients seen daily may not reach this number in some areas (although it may also exceed it in others). An emerging contradiction is that HCPs appear to believe that they are referring an ‘appropriate’ amount. For example, Participant 7 referred patients to SP just twice per month, despite their positivity about the service. When was asked what (if anything) would increase their engagement with SP, they answered that nothing would: ‘*I feel I am using the service appropriately already’*. Furthermore, one GP referred to themselves as a *‘high user*’ of SP, but again they were referring patients approximately twice per month. Such results do not explain why practitioners refer so rarely, although one explanation is provided by Participant 4 who reported that they would engage more with SP if there was *‘a SP with larger capacity*’. Overall, findings within this domain indicate a reluctance to use SP to its full extent, while recognising the caveat that the NHS LTP is indeed long term in nature and was only 2 years into implementation at the time of the survey.

**TABLE 2:** Self‐reported frequency of referrals to social prescribing

Frequency of referrals	Number of respondents
2–3 times per week	3
Once per week	3
Once per fortnight	7
Once per month	5
Less often	5

An additional finding in this domain was that 23/24 (95.8%) reported directly signposting patients rather than referring them to SP (for example, to weight loss, alcohol and drug services, Citizens Advice, well‐being hubs, self‐help websites and charities). This serves to demonstrate engagement with the SP belief that patients with non‐medical needs should be linked with non‐medical sources of support. This would fit with one of Kimberlee's (2015) four models of SP—‘signposting’—which is the lowest‐intensity model. Therefore, HCPs may have been engaging a one model of SP (signposting) since before the introduction of SP into the NHS remit. Nevertheless, this high community referral rate would not be reflected in SP statistics, as only formal SP referrals are digitally recorded and are visible on national reports. Therefore, HCPs are practicing SP through an existing, informal pathway rather than the channel created by the NHS model of SP, even if they do not name it as such. This research was carried out during the Covid 19 pandemic, though no evidence emerged indicating that HCPs were referring to SP less as a result of any Covid‐19 restrictions.

### 3.5 Relationships with link workers

This domain relates to how HCPs perceive their relationships with SP link workers. This relationship is a vital link in the chain of SP which, as discussed, has been emphasised in previous evidence. There is a clear dichotomy in experience of HCPs in the locality investigated, as reflected between the themes ‘close links and communication with SP team’ and ‘more communication and interaction needed’. The former includes reports of link workers attending multi‐disciplinary team meetings, working tightly together and having close relationships. Participant 11 described their local SP services as a ‘*brilliant local team… motivated and skilled’*, and Participant 4 reported ‘*a great local service forming and delivering at pace*’. In contrast, the latter comprised of sub‐themes such as ‘would like more interaction with link workers’ and ‘would like better communication with SP team’. Within this sub‐theme were responses such as ‘*our previous social prescriber wasn't available very much so there was a backlog of referrals. She may have been on sick leave’* (Participant 6); and calls for *‘more interaction for SP team‐ named worker etc’* (Participant 1), ‘*greater presence in the practice’* (Participant 19), ‘*more engagement from the social prescribing link worker’* (Participant 6), and ‘*being able to have more direct access/communication with the involved SP’* (Participant 15).

Participants were asked if they knew the name of their attached SP link worker and whether they were ever based in the GP surgery. Of the 24 respondents, 16 reported to know the name of their link worker. The amount of time the link worker was thought to be based in the surgery varied between ‘unsure’ (11), ‘never’ (2), ‘rarely’ (2), ‘occasionally’ (5) and ‘often’ (4). No HCPs reported link workers working ‘full time’ in their surgeries. Future phases of this research will aim to ascertain the accuracy of this reporting, particularly whether link workers remain ‘unseen’ by HCPs yet are based in the surgeries more regularly in reality. The issue of link worker visibility has been discussed in prior evidence, with South et al. (2008) reporting that health professionals were more comfortable making referrals to link workers they knew and in whom they had higher levels of trust. Additionally, Fixsen et al. (2020) found that maintaining a visible presence in practices was effective for promoting practitioner buy‐in, although referrals slowed as soon as the link worker was no longer regularly in the practice. This research did not ascertain whether link workers have never been based in surgeries, or if the Covid‐19 pandemic has negatively impacted this visibility because link workers may have been working from home or have reduced their interaction within workplaces (such as shut doors and staggered lunch breaks). This question will be explored in the planned second study within this research project which will comprise interviews with link workers themselves. These responses demonstrate that, within this CCG at least, clear relationships between link workers and primary care teams have not always been adequately established.

## 4 DISCUSSION

HCPs report that their knowledge and understanding of SP has grown and developed in the preceding 2 years since the publication of the NHS LTP and introduction of an NHS model of SP. Results of our survey indicate increasing engagement with the practice which can almost certainly be attributed to it being brought firmly within the NHS remit. This finding may also be due to the increasing influence of the biopsychosocial model of health within medical or healthcare education and training, which emphasises more non‐medical interventions and addressing patients' social as well as medical needs (Farre & Rapley, 2017). Evidence for this appeared in the apparent link between the amount of time an HCP had been practicing and their confidence in their understanding of SP. The increasing engagement with SP may indeed be a combination of these factors. Factors reported to have aided this increased understanding included the provision of case studies and an SP newsletter.

This knowledge does, nonetheless, remain inconsistent. HCPs define the practice in numerous different ways and provide varying reasons for referral. A number reported that they would engage more with SP if they knew more about the role, who would be suitable for referral and how SP could help. This chimes with previous literature which emphasised the importance of promoting HCP buy‐in to SP schemes (Bickerdike et al., 2017; Fixsen et al., 2020). Despite this inconsistency, perceptions of SP are predominately positive within the HCP cohort studied. Significantly, this positivity regarding SP does not seem to have translated into a wealth of referrals, as most HCPs reported referring a patient approximately once per fortnight or less frequently. The reason for the discrepancy between high positivity of SP and low rates of referral is not clear through this study and warrants further investigation in future research.

Inconsistency was again prevalent in referral reasons, as well as in referral and feedback practices. HCPs often referred patients for practical reasons. This is significant as practical assistance is one of the aims of SP, yet previous literature has failed to address it fully. Referral processes themselves varied from prepopulated electronic forms and a clear referral procedure to opinions that the forms were time‐consuming and confusing. Self‐referrals were thought to be permitted in some GP surgeries and not in others. In some cases, GP administrators could refer patients to SP at the point of contact. No clear adherence to an NHS model of practice was found, although it is acknowledged again that the NHS model is in the process of integration into primary care. Variations in practices may be a ‘cultural’ legacy of established or previous bottom‐up schemes which have not yet transitioned into a standardised NHS practice. The Covid‐19 pandemic additionally may be thought to have impacted upon establishing of a new NHS model, yet no evidence for this was found within participant responses.

The relationships between HCPs and SP teams showed considerable variation throughout the CCG investigated. Some participants were highly positive of SP link workers, but others could not name their link worker and were unsure if they had ever been based within the GP surgery. This finding requires further examination, especially with regards to the factors which facilitate the establishment of close relationships. It is important because closer, trusting relationships between primary care and SP teams have been previously thought to be beneficial to referral rates and to facilitate good SP practice.

## 5 CONCLUSION

This study provides insights into HCP perceptions of SP within one CCG. It indicates a developing understanding of, and engagement with, SP since the publication of the NHS model (NHS England, 2019b). However, results demonstrate variation in practice at every stage of SP, including the definitions provided, reasons for referrals, referral and feedback processes and the relationships HCPs have with link workers. Factors promoting the integration of the NHS model to primary care are; education regarding SP, learning from case studies, information via newsletters and guidance on more uniform, integrated and standardised referral and feedback practices. More research is recommended regarding link workers' relationships with HCPs and engagement with practices, whether link workers are predominately engaging in providing practical assistance, whether referral processes elsewhere are as varied as reported in this study, and the associations between HCPs positivity towards the SP model and referral rates. Additionally, this research advocates for increased education and CPD for HCPs on all aspects of SP including theory and practice, and intentional time and space provided for this education, along with opportunities for HCPs, link workers and SP providers to forge greater connections and stronger links.

## AUTHOR CONTRIBUTIONS

C.M conceptualised and designed the research with support and supervision from P.E, N.E and F.H. C.M carried out all interviews, transcription and thematic analysis of data. C.M drafted the manuscript. All authors discussed results and commented on the manuscript. P.E, N.E and F.H edited the manuscript and provided expert guidance.

## FUNDING INFORMATION

This research was funded through a University of Worcester PhD studentship.

## CONFLICT OF INTEREST

Dr Frances Howie is currently employed as a public health consultant in one of the counties where the study took place and was instrumental in the development of the social prescribing pilot, which was part‐funded by the Public Health ringfenced grant.

## Data Availability

The data that support the findings of this study are available from the corresponding author, CM, upon reasonable request.
